# Adherence to the National Tobacco-Free School Policy in Selected Schools of Puducherry District in India: A Cross-Sectional Exploratory Study

**DOI:** 10.7759/cureus.53984

**Published:** 2024-02-10

**Authors:** Shubhajit Pahari, Parthibane Sivanantham, Sitanshu S Kar

**Affiliations:** 1 Department of Preventive and Social Medicine, Jawaharlal Institute of Postgraduate Medical Education and Research, Puducherry, IND

**Keywords:** adolescents, students, tobacco-free educational institutions, the cigarettes and other tobacco products act (cotpa) 2003, smoking, smokeless tobacco, rural health, schools

## Abstract

Introduction: The objective of this study was to estimate the level of compliance and the factors associated with high adherence to the Tobacco-Free Educational Institutions (ToFEI) guidelines of the Government of India among schools in the district of Puducherry, India.

Methods: This cross-sectional study was conducted among schools (N=50) in the Puducherry district in 2021-2022 using a "Self-Evaluation Scorecard" of the ToFEI guidelines. The assessment was done through in-person interviews with the schools' heads/representatives. The level of compliance to indicators was presented as proportions, and factors associated with high compliance were assessed using the chi-square test.

Results: No school met all the ToFEI indicators. The majority (88%) showed no evidence of the use of tobacco products inside the premises. More than half of the schools (58%) adhered to the criteria of not having tobacco shops within 100 yards and 56% reported the inclusion of the “No Use of Tobacco” norm in their guidelines. Schools located in rural areas (p-value <0.01) and those with teachers who attended any tobacco-related workshop were more likely to comply with the ToFEI indicators (p-value 0.05). After relaxing the criteria for ‘High Adherence’ to at least four indicators, we found that 20% of schools showed high adherence to the ToFEI indicators.

Conclusion: Overall compliance of schools to the ToFEI guidelines is low in Puducherry. Sensitizing the relevant stakeholders in the district for implementing ToFEI guidelines and institutionalizing tobacco control activities in the school are the needs of the hour.

## Introduction

Tobacco use is one of the biggest public health threats that countries worldwide have ever faced [1]. Globally, tobacco kills about eight million people, of which seven million deaths are due to tobacco use, and about 1.2 million are due to passive smoking [2]. Globally, about one-third (32.7%) of men and about 6.62% of women aged ≥15 years use tobacco [3]. Smoking tobacco is a significant cause of cardiovascular and respiratory diseases, cancer, and a variety of other debilitating health conditions [4].

Among the youth, tobacco use is increasing in epidemic proportion globally [5]. In India, around 5-25% adolescents use or have ever used tobacco. High rates of smokeless tobacco use have been reported among adolescents aged 13-15 years (15% of boys and 5% of girls) [6]. In Puducherry, 11.2% of people ≥15 years use tobacco in one form or the other, with men and women accounting for 17.7% and 5.1%, respectively [7]. According to the Global Youth Tobacco Survey (GYTS), 2019, 8.5% of students, 9.6% of boys, and 7.4% of girls currently use some form of tobacco products in India [8].

Adolescents are the crucial market because they are unaware of the enticing tactics imposed by the tobacco industry and lack the skills to deter themselves from unhealthy habits [9]. In India, about 20 million children aged 10-14 years are tobacco addicts. Every day in India, about 5500 adolescents begin smoking, bringing the total number of new smokers to two million per year [10]. Considering the significant burden of tobacco use in the country, the Government of India enacted the Cigarette and Other Tobacco Products Act (COTPA) in 2003, which prohibits advertising and regulates the trade, manufacturing, supply, and distribution of cigarettes and other tobacco products in India. In particular, to deter tobacco use among adolescents, the National Tobacco Control Programme (NTCP) of India developed guidelines for “Tobacco-Free Educational Institutions (ToFEI),” which have to be followed by educational Institutions across the country [11]. A study in Spain showed that adolescent smoking was significantly reduced when school-centered prevention and control programs were implemented [12]. A study in Bihar, India, also showed that implementing tobacco-free policies in schools is associated with reduced tobacco use among students and teachers [13].

In line with this evidence, establishing tobacco-free educational institutes in Puducherry has been one of the key activities under the NTCP since its launch in 2016 [14]. However, few studies have been conducted so far to assess the compliance status of tobacco-free educational institutes in Puducherry. Therefore, we undertook this study to estimate the level of compliance and the factors that influence adherence to ToFEI guidelines across schools in the district.

## Materials and methods

A cross-sectional exploratory study was conducted from June 2021 to May 2022 among schools located in the district of Puducherry, located in the union territory of Puducherry, in India's southeastern coast, bordered by the state of Tamil Nadu.

The total population of the Puducherry district is 0.95 million with the majority residing in urban areas (69.2%) [15]. Puducherry has a sex ratio of 1112, a literacy rate of 85.85%, and a human development index of 0.740 [16]. In the district, 20.7% and 19.7% of the population in urban and rural areas, respectively, are below 15 years of age [17]. Rural areas of the Puducherry district are divided into five communes: Ariyankuppam, Villianur, Mannadipet, Bahour, and Nettapakkam, and the urban areas are divided into two municipalities: Puducherry and Ozhukarai. There are around 500 schools in the district. Among them, 277 and 220 are government and private-owned, respectively [18].

NTCP was launched in Puducherry district in 2016. One of the main activities of NTCP is implementing anti-tobacco related activities in schools such as training teachers on the prevention and control of tobacco use, providing information, education, and communication (IEC) materials, conducting awareness campaigns, enforcing and monitoring the implementation of sections 4 and 6 of the COTPA, and setting up tobacco control committees across schools in the district. In 2020, NTCP started a “Yellow Line Campaign” in the district to demarcate a 100-yard circular area around each educational institute as a tobacco-free zone in which shopkeepers were sensitized to avoid selling tobacco products as per Section 6 (b) of COTPA, 2003.

It should be noted that this study was conducted amid the coronavirus disease 2019 (COVID-19) pandemic; hence, all the schools were not fully functioning and many shops in and around schools remained closed due to government restrictions.

Sample size and sampling

**Figure 1:**
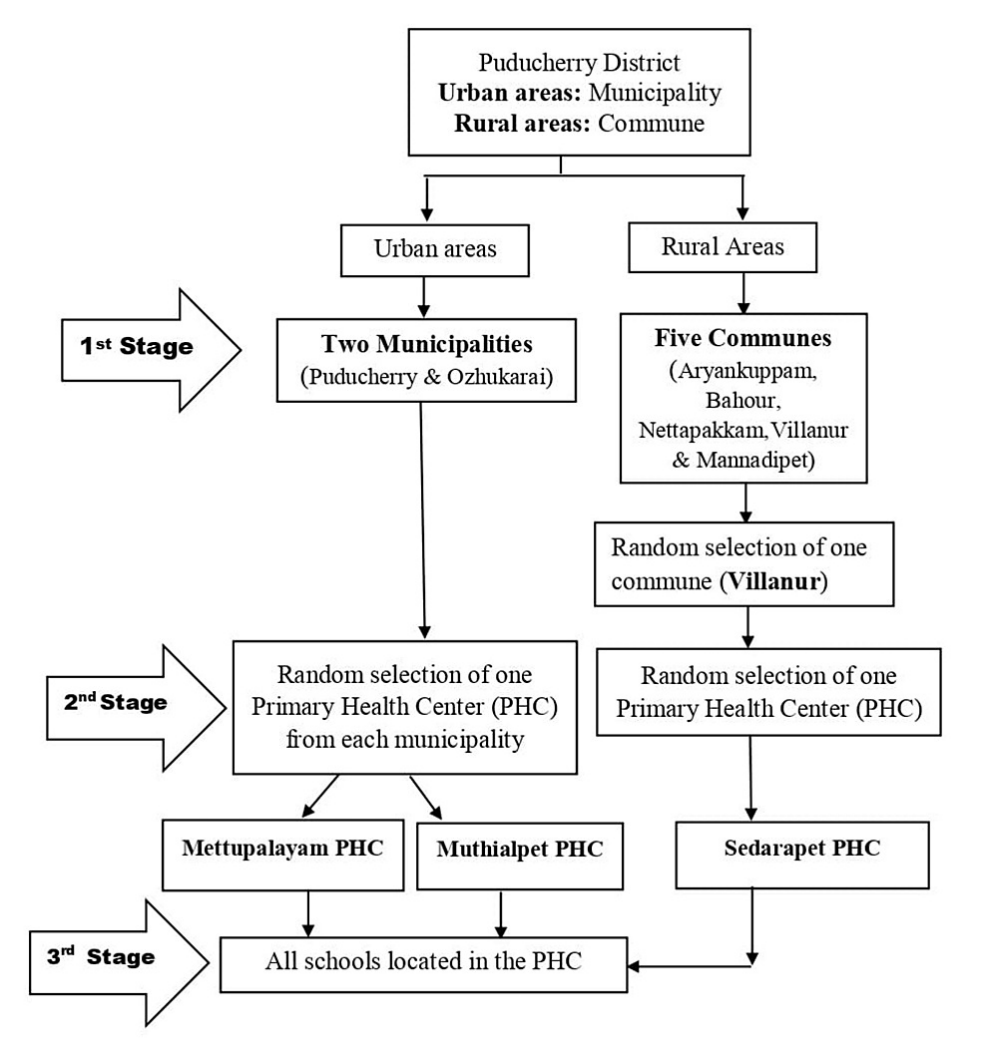
Flow diagram showing the sampling procedure used for the study

Considering the exploratory nature of the study, available resources, and feasibility, the sample size was not calculated. For the study, we chose both the district municipalities (Puducherry and Ozhukarai), and out of five communes, we chose one commune (Villanur) randomly. Of the three study clusters selected, we initially listed all the Primary Healthcare Center (PHC) service areas. From each cluster, one PHC service area (Muthialpet, Mettupalayam, and Sedarapet) was selected through simple random sampling. There were 55 schools (Primary/ Middle/ Secondary/Higher Secondary) in the selected PHC service areas, and all of them were included in the study. The study process is illustrated in Figure 1.

Study tool

We adopted the tool from the ToFEI guidelines prescribed by the Ministry of Health and Family Welfare (MoHFW), the "Self-Evaluation Scorecard", for assessing the schools' adherence to ToFEI indicators [11].

This self-evaluation scorecard lists nine indicators of which the first three are categorized as “mandatory”: (i) the existence of "Tobacco-free Zone" signage within the educational institutes' premises (score 10) with the name/designation/contact number of the responsible authority (score 10), (ii) display of such signage at the entrance or boundary wall of EI (score 10) with the name/designation/contact number of the responsible authority (score 10), and (iii) no indication of tobacco use on campus (score 10). Other indicators include: the presence of awareness material on the harms of tobacco in the premise, organization of tobacco control activities, inclusion of “No-Tobacco Use" norms in the institute's code of conduct regulations, marking of a 100 yards area around the outer limit of the boundary wall of the school and a ban of selling tobacco within this area.

Every criterion has its weightage points. As per the guidelines the first three mandatory criteria carry a maximum total score of 50. The following four criteria carry a score of 9 each and the last two criteria carry a score of 7 each. The maximum total score that a school could achieve is 100.

Data collection

A single investigator was involved in data collection from July 2021 to December 2021 using Epicollect5 (Centre for Genomic Pathogen Surveillance, Oxford, United Kingdom). Coordinates of the schools and tobacco shops were collected through Mobile Topographer (S.F. Applicality Ltd., Athens, Greece). In-person visits to schools were made during school hours. The assessment was done after describing the study procedure, objective, and outcomes, and consent was taken from the head of the school. Initially, we collected information on the sociodemographic characteristics of schools by interviewing the head of the school or the assigned school representative. Adherence to ToFEI such as posters and signage and maintaining a copy of COTPA law were assessed using the self-evaluation scorecard by interviewing the participant and observing within the school premises. Every school was contacted twice for participation. If no response was received, the school was excluded from the study.

Operational definitions

High level of adherence indicated adherence with ≥ 90% of ToFEI indicators, i.e. ≥8 indicators. Low level of adherence indicated adherence to less than eight indicators.

Statistical analysis

Analysis was done using IBM SPSS Statistics for Windows, Version 22.0 (Released 2013; IBM Corp., Armonk, New York, United States). Categorical variables such as sociodemographic characteristics and the level of adherence to the nine ToFEI indicators were expressed as proportions. Continuous variables were expressed as median (Interquartile range (IQR)) depending upon normality. Factors associated with school-level indicators were assessed using Pearson’s Chi-squared (χ2) test. Depending on the number of groups and normality of distribution of outcome variable, the comparison of mean/median scores between groups was assessed using t-test/Mann-Whitney U and ANOVA/Kruskal-Wallis test. In the analysis, a p-value of ≤0.05 was considered statistically significant. To assess criteria No. 9 (“No shops selling tobacco products within 100 yards of the educational institute), buffer analysis was done in ArcGIS Pro 2.7 (2020; Environmental Systems Research Institute, Inc., Redlands, California, United States).

## Results

The total number of schools approached was 53. The response rate of schools was 94.3% (50/53). More than half the schools (n=29, 58%) were government-managed and a majority (n=40, 80%) were situated in urban areas and co-educational (n=42, 84%). Over four out of five schools conducted cultural events (n=44, 88%) and sports (n=41, 82%) (Table 1).

**Table 1:** Sociodemographic characteristics, facilities, and extracurricular activities of schools included in the study (N=50)

Variables	Number of schools (n)	Percentage (%)
Type of schools		
Government managed	29	58
Private managed/aided	21	42
Location of schools		
Rural	10	20
Urban	40	80
Gender composition of schools		
Boys only	4	8
Girls only	4	8
Co-educational	42	84
Type of building		
Kutcha	0	0
Pucca	50	100
Medium of instruction		
English	47	94
Regional language	3	6
Number of classrooms		
1-5	6	12
6-9	19	38
10 and above	25	50
School facilities		
Toilets	50	100
Parent-teacher association	49	98
Playground	28	56
Internet connectivity	23	46
E-learning center	21	42
Extracurricular activities		
Cultural events	44	88
Sports	41	82
School teachers attended tobacco-related workshops	16	32
Receipt of government awards by school	13	26

As none of the surveyed schools adhered to all the nine indicators of ToFEI, we relaxed the criteria for "High level" of adherence. If any school adhered to at least four indicators, then the school was regarded as having a high level of adherence. To assess adherence according to the modified criteria, we derived a composite score by assigning a score of one for adherence to each indicator. Therefore, a school could score a maximum score of 9. We found that the mean composite score for schools was 2.50 (±1.34) indicating that on average, schools adhered to two to three indicators prescribed in the ToFEI guidelines.

Based on the modified criteria, only about 10 (20%) schools showed high adherence to the ToFEI indicators. In this study, no school met all the ToFEI indicators. Most of the schools (n=44, 88%) adhered to the indicator 3 (No evidence of use of tobacco products inside the premise). Over half of the schools reported having “No Use of Tobacco” norm in their code of conduct guidelines (n=28, 56%) and adhered to the criteria of not having tobacco shops within 100 yards of the institute (n=29, 58%) (Table 2).

**Table 2:** Level of adherence to the Tobacco-Free School (TFS) criteria by schools in the study (N=50) TFS: Tobacco-Free School

S. No	Indicators of TFS criteria	Schools that met TFS Criteria, n (%)
1.	Display of “Tobacco-Free Area” signage inside the premises of the educational institute at all prominent places. The name /designation/contact number is mentioned/updated in the signage (Mandatory).	5 (10%)
2.	Display of “Tobacco-Free Education Institution” signage at the entrance/ boundary wall of the educational Institute. The name/designation/contact number is mentioned/updated in the signage (Mandatory).	1 (2%)
3.	No evidence of the use of tobacco products inside the premise, i.e. cigarette/beedi butts or discarded gutka/tobacco pouches (Mandatory).	44 (88%)
4.	Poster or other awareness materials on the harms of tobacco displayed in the premise.	3 (6%)
5.	Organization of at least one tobacco control activity during the last six months.	11 (22%)
6.	Designation of Tobacco Monitors and their names, designations, and contact numbers mentioned on the signages.	2 (4%)
7.	Inclusion of “No Tobacco Use” norm in the EI’s code of conduct guidelines.	28 (56%)
8.	Marking of 100 yards area from the outer limit of boundary wall/fence of the educational institute.	2 (4%)
9.	No shops selling tobacco products within 100 yards of the educational institute.	29 (58%)

Among the schools surveyed, the schools located in the rural areas (prevalence ratio (PR) 2.66) and schools whose teachers have attended any tobacco-related workshop (PR 3.08) had significantly higher levels of adherence to ToFEI guidelines when compared to their counterparts (p-value ≤0.05) (Table 3).

**Table 3:** School-level facilities and extracurricular activities associated with the adherence to the Tobacco-Free School indicators for the schools included in the study (N=50) *p-value calculated by chi-square test. p-value ≤0.05 statistically significant

Variables	Number of schools (n)	Low adherence, n (%)	High adherence, n (%)	Prevalence ratio (PR)	p-value^*^
Type of schools					
Government managed	29	24 (82.8%)	5 (17.2%)	1	0.72
Private managed/Aided	21	16 (76.2%)	5 (23.8%)	1.41	
Location of the schools					
Rural	10	6(60)	4(40)	2.66	<0.01
Urban	40	34(85)	6(15)	1	
Gender composition in schools					
Co-educational	42	34(81)	8(19)	1	0.65
Others	8	6(75)	2(25)	1.31	
Number of classrooms					
1-5	6	5(83.3)	1(16.7)	1.06	0.77
6-10	19	16(84.2)	3(15.8)	1	
More than 10	25	19(76)	6(24)	1.5	
Playground					
Yes	28	21 (75)	7 (25)	1.78	0.48
No	22	19 (86.4)	3 (13.6)	1	
Internet connectivity					
Yes	23	16(69.6)	7(30.4)	2.72	0.15
No	27	24(88.9)	3(11.1)	1	
E-learning center					
Yes	21	14(66.7)	7(33.3)	3.26	0.07
No	29	26(89.7)	3(10.3)	1	
Parent teacher association					
Yes	49	40(81.63)	9(18.36)	1	1.0
No	1	0	1(100)	-	
Sports					
Yes	41	33(80.5)	8(19.5)	1	0.17
No	9	7(77.8)	2(22.2)	1.1	
Cultural events					
Yes	44	36(81.8)	8(18.2)	1	0.58
No	6	4(66.7)	2(33.3)	1.83	
School teacher attended tobacco related workshop					
Yes	16	10(62.5)	6(37.5)	1	0.05
No	34	30(88.2)	4(11.8)	3.08	

Among the schools surveyed, the median overall ToFEI scores for rural and urban schools were 32.5 (IQR 9.75-40.75) and 19 (IQR 17-26), respectively. For rural schools, the score ranged from 7 to 79 and for urban schools, it ranged from 0 to 56. Only one school in a rural area achieved the maximum mandatory score, i.e. 50. The median overall scores were higher among the private schools compared to their counterparts.

**Figure 2:**
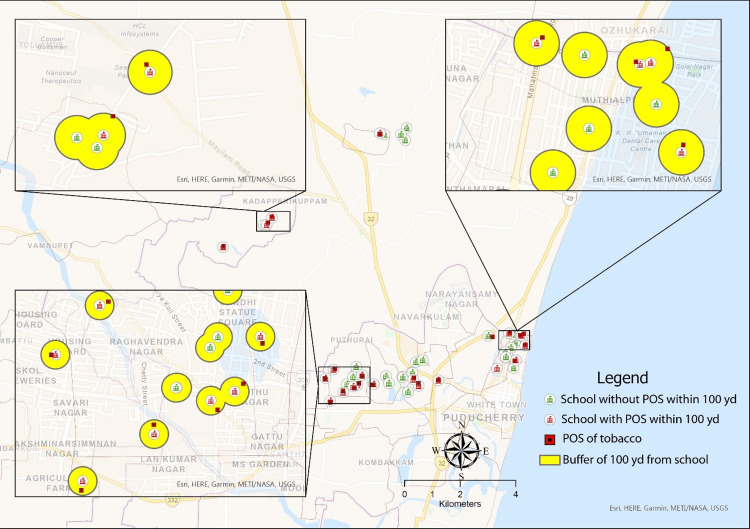
Points of sale of tobacco products around the schools included in the study (N=50) POS: point of sale

In the study, almost four out of 10 schools, i.e., 21 (42%) had points of sale (PoS) within a 100-yard radius. Tobacco shops were at distances as short as 15.05 yards (13.77 meters) and as far as 96.13 yards (87.91 meters) from the boundaries of the surveyed schools. Among the schools that had PoS within 100 yards, the average distance between the school and PoS was 51.61 yards (47.2 meters) (Figure 2).

## Discussion

In this study, no school adhered to all the nine indicators of ToFEI. Consistent with the current study findings, a study conducted in Haryana, India, in 2020 also found that none of the schools adhered to all the ToFEI indicators [19]. In contrast, studies conducted in rural parts of Maharashtra and Chandigarh in India found that 11% and 37.5% of schools adhered to all the ToFEI indicators, respectively [20,21]. The differences observed in the compliance with ToFEI indicators could be attributed to differences in the level of implementation of COTPA across states in the country. In particular, a significantly higher level of compliance to ToFEI indicators in Chandigarh (37.5%) could be attributed to various long-standing, concerted tobacco control measures implemented by the Chandigarh Tobacco Control Cell through multi-sectoral cooperation, making it the country's first smoke-free city in 2007 [22].

Studies conducted in low and middle-income countries (LMICs) found that students who smoked on school premises are at increased risk of becoming smokers and in a study in Sudan, more than half of the study participants reported nonexistent TFS policies [23]. Similar to the present study's findings, evidence suggests that non-compliance to TFS policies is widespread in LMICs [24]. Therefore, it is critical that stakeholders in LMICs should take the initiative and implement effective measures to increase the compliance level. This emphasizes that the tobacco control managers of India and other countries undertake research to generate baseline data on this, which is crucial in preventing and controlling tobacco use among school-aged children.

In this study, almost half (42%) of the schools showed evidence of violating section 6(b) of COTPA. A study conducted in 307 schools across five states in India (Andhra Pradesh, Uttar Pradesh, Odisha, Jharkhand, and Karnataka) also found violations of section 4 (85%) and 6b (69%) of COTPA to a larger extent [25], when compared to the current study. Comparably better compliance in the present study could be due to various school-level anti-tobacco activities undertaken by the tobacco control cell of the Puducherry district over the last few years, especially the "Yellow Line Campaign", which increased the section 6 (b) compliance in the district since 2020.

A study conducted in China in 2005 and another study conducted in Spain in 2012 discovered that the implementation of smoking-related educational programs decreased the smoking prevalence in schools [26,12]. In light of the above-cited evidence, we highlight that adherence to ToFEI guidelines will curb the use of tobacco products in the district of Puducherry by making school premises tobacco-free. Effective implementation of such programs in LMICs like India will help in the efforts towards prevention and control of tobacco use among school-aged children in such countries.

In this study, only 10% of schools had a “Tobacco-Free Area” board inside the campus and almost no schools had a display of “Tobacco Free Education Institution” signage at the entrance/boundary wall, which is almost similar to the study conducted in Haryana in 2021 (5.9% and 2.4%, respectively) [19]. In contrast, a study conducted in rural areas of Maharashtra in 2017 reported almost 31% of schools had banners, posters, etc. at the entrance of schools and almost 45% inside the premises. A study conducted in Chandigarh also showed around 60% of educational institutes and 38% in Delhi had signage of tobacco-free zones [21,27]. Concerningly low compliance in the current study compared to other studies suggests that state tobacco control managers and stakeholders should come up with strategies to raise these tobacco-free indicators across all educational institutions in the district. In this regard, the NTCP of Puducherry should work with others to establish strategies to improve compliance. For instance, joint initiatives such as issuing circulars mandating the display of required signages in schools, supplying these boards to schools by the district NTCP cell, and conducting sensitization programs could be crucial in improving the compliance level of schools on these tobacco-free indicators.

In this study, one-fifth (22%) of schools engaged in at least one tobacco control activity in the previous six months contrasting the result of the study done in Haryana in 2021, where no school conducted any tobacco control activity during the past six months [19]. Although compliance with this indicator is comparatively better in Puducherry, the level of compliance on this indicator is still low, and this may also be attributed to the COVID-19 situation in the country at the time of the study when almost all schools in Puducherry remained closed, making conducting anti-tobacco activities redundant.

In this study, only one school achieved mandatory (i.e. 50), and no school achieved overall (i.e. 100) scores of ToFEI guidelines. This finding indicates an overall lack of adherence in both government and private schools across rural and urban areas. The government and relevant stakeholders should identify and implement successful strategies to institutionalize ToFEI guidelines across educational institutes in the district. For instance, in the year 2010, the neighboring state of Tamil Nadu declared 2000 educational institutes tobacco free in five months. This feat was achieved through an innovative campaign launched in collaboration with an NGO to establish smoke-free educational institutes in the state [28]. The campaign included constituting an anti-tobacco cell, conducting sensitization workshops, and issuance of guidelines for compliance across schools in the state.

In the current study, we found that schools where teachers had attended anti-tobacco-related programs had more adherence to ToFEI. A study conducted in China revealed that interventional anti-tobacco programs could enhance students' refusal skills toward smoking [26]. This suggests that the NTCP of Puducherry should conduct more teacher training and sensitization programs to promote a tobacco-free environment in schools. A study conducted in rural schools in Maharashtra in 2017 showed schools' participation in sports, availability of internet connectivity, e-learning facilities, etc. have a significant association with the adherence to ToFEI guidelines [20]. This indicated that adequate physical activity with the easy accessibility of knowledge (with the help of the internet) helped students, teachers, and other school personnel to be deterred from tobacco-related behaviors. In our study, rural schools adhered more to the ToFEI guidelines than urban. This observation could be because schools in urban areas are more susceptible to violating the regulations as the areas are densely populated, presenting a good opportunity for business and advertisements of tobacco products. This needs more attention and strict vigilance by the government administration and the school authorities so that the schools in this area will show more adherence to ToFEI guidelines.

Limitations

The level of adherence to ToFEI indicators was assessed from the self-report of designated school representatives. Therefore, some level of social desirability bias could have influenced the level of adherence to various ToFEI indicators estimated in the study. To minimize this bias, the investigator undertook an inspection of the school after the interview, to verify the responses. We conducted this study during the COVID-19 pandemic when varying levels of COVID-19-associated restrictions were in place. As a result, a large number of PoSs around the schools remained closed. This made it difficult for the investigator to evaluate the schools' compliance with Section 6(b) of the COTPA.

## Conclusions

Schools in Puducherry have a low level of adherence to the ToFEI indicators. In particular, no school adhered to all the nine indicators of ToFEI, almost half of the schools had a PoS within 100 yards of the school, only one out of 10 schools had a “Tobacco-free Area” board inside the campus and almost no school displayed “Tobacco-Free Educational Institution” signage at entrance/boundary wall. We emphasize that government policy and school-level tobacco control initiatives such as constituting field-level compliance assessment squad, sensitizing school authorities and teachers on tobacco control, and institutionalizing tobacco control activities in schools, could help to prevent and control tobacco use as well as improve the school’s adherence to ToFEI guidelines in the district.
